# Comprehensive Insights into Highly Pathogenic Avian Influenza H5N1 in Dairy Cattle: Transmission Dynamics, Milk-Borne Risks, Public Health Implications, Biosecurity Recommendations, and One Health Strategies for Outbreak Control

**DOI:** 10.3390/pathogens14030278

**Published:** 2025-03-13

**Authors:** Henrietta Owusu, Yasser M. Sanad

**Affiliations:** 1 Department of Agriculture, University of Arkansas, Pine Bluff, AR 71601, USA; 2 Department of Epidemiology, University of Arkansas for Medical Sciences, Little Rock, AR 72005, USA

**Keywords:** highly pathogenic avian influenza (HPAI), dairy cattle infections, milk-borne transmission, zoonotic influenza, one health approach, biosecurity measures, public health risk

## Abstract

Highly pathogenic avian influenza (HPAI) H5N1 has been traditionally linked to poultry and wild birds, which has recently become a serious concern for dairy cattle, causing outbreaks all over the United States. The need for improved surveillance, biosecurity protocols, and interagency collaboration is highlighted by the discovery of H5N1 in dairy herds in several states and its human transmission. The epidemiology, transmission dynamics, and wide-ranging effects of H5N1 in cattle are reviewed in this paper, with particular attention paid to the disease’s effects on agricultural systems, public health, and animal health. Nonspecific clinical symptoms, such as decreased milk production and irregular milk consistency, are indicative of infection in dairy cows. Alarmingly, significant virus loads have been discovered in raw milk, raising worries about potential zoonotic transmission. The dangers of viral spillover between species are further highlighted by cases of domestic cats experiencing severe neurological symptoms after ingesting raw colostrum and milk from infected cows. Even though human cases remain rare, and they are mostly related to occupational exposure, constant attention is required due to the possibility of viral adaptability. The necessity of a One Health approach that integrates environmental, animal, and human health efforts is further supported by the broad occurrence of H5N1 across multiple species. For early detection, containment, and mitigation, cooperation between veterinary clinics, public health organizations, and agricultural stakeholders is crucial. Controlling the outbreak requires stringent movement restrictions, regular testing of dairy cows in reference labs, and adherence to biosecurity procedures. This review highlights the importance of thorough and coordinated efforts to manage H5N1 in dairy cattle by combining existing knowledge and pointing out gaps in surveillance and response strategies. Additionally, it sheds light on the potential risk of consumption of cow’s milk contaminated with H5N1 virus by humans and other companion animals like cats. In the face of this changing threat, proactive monitoring, strict biosecurity protocols, and cross-sector cooperation are crucial for reducing financial losses and protecting human and animal health.

## 1. Introduction

Avian influenza (AI), originated from the animal reservoir, is a significant current public health issue [1]. Influenza viruses are segmented, negative-strand RNA viruses belonging to the *Orthomyxoviridae* family, which includes three genera: influenza virus A, B, and C. Avian influenza is a type A influenza virus, specifically adapted to an avian host. The virus is enveloped and pleiomorphic, with sizes ranging from 80 to 120 nm [2]. The hemagglutinin (HA) protein has 16 subtypes (H1-H16) that include neutralizing epitopes. In contrast, antibodies to the neuraminidase (NA) are not neutralizing, and there are nine neuraminidase subtypes (N1-N9). All 16 HA subtypes have been found in ducks, gulls, or shorebirds, the natural reservoir hosts of the virus. Certain subtypes are more prevalent in specific species; for instance, H3, H4, and H6 are common in North American ducks. While there is no strict association between host range or host restriction based on HA subtypes, some subtypes are more frequently found in particular species such as H1 and H3 in swine, H3 in horses, and H5 and H7 in chickens [3]. In March 2024, it was reported that the A (H5N1) virus infected dairy cows in the United States, with infections likely beginning in December 2023. As of 13 January 2025, the Centers for Disease Control and Prevention (CDC) reported that all 50 states in the United States have recorded H5N1 outbreaks with a total outbreak of 1410 cases in poultry and wild birds in 606 counties [4]. There have been 53 human cases of H5 bird flu in the United States, with 24 cases associated with exposure to sick or infected dairy cows [5]. The majority experienced symptoms of eye irritation, such as redness and discharge, while a smaller number reported mild respiratory symptoms. Many cases have been concentrated in California and Colorado, indicating potential regional patterns that may lead further investigation into transmission routes and risk factors [5]. The cattle exhibited nonspecific signs such as decreased milk production, reduced rumination, and thickened, colostrum-like milk; some also showed clear nasal discharge. High levels of the A(H5N1) virus were detected in unpasteurized (raw) milk from affected animals [6]. Domestic cats fed raw colostrum and milk from sick cows in the hospital parlor on more than one dairy farm in Texas died. Depressed mental state, rigid body movements, ataxia, blindness, circling, and copious oculo-nasal discharge were the antemortem clinical signs observed in the affected cats. The affected cats’ neurologic examinations showed weak blink responses and no menace reflexes or pupillary light responses [7].

These cases underscore the occupational risks associated with animal handling in agricultural settings and highlight the importance of protective measures to minimize transmission risks from animals to humans [8]. Close monitoring and timely reporting of such cases are essential for early detection and containment, especially given the potential for zoonotic influenza viruses to adapt and pose broader public health threats. This extensive spread underscores the importance of heightened biosecurity measures, routine testing, and interagency coordination to control the spread of avian influenza among dairy cattle populations, minimizing both economic losses and public health risks.

## 2. Epidemiology and Transmission

Recent outbreaks have brought considerable attention to the epidemiology and transmission dynamics of HPAI H5N1 in dairy cattle [8]. In the past, H5N1 mostly afflicted birds, but starting April 2024, cases have been reported in dairy cattle in several U.S. states, suggesting a significant change in the virus’s host range. The B3.13 clade is the most common strain, and as of December 2024, approximately 950 herds across 16 states had been impacted [9].

**Figure 1:**
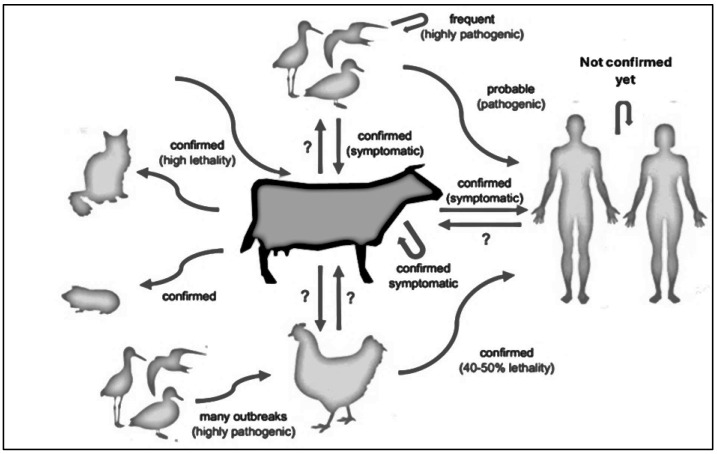
Transmission dynamics of HPAI H5N1. Arrows represent confirmed and possible transmission pathways across species. The diagram illustrates frequent and severe outbreaks in birds, documented symptomatic cases in cattle, and the potential, yet unconfirmed, risk of transmission to humans.

Investigations are still ongoing to determine the precise pathways that allow H5N1 to spread to dairy animals (Figure 1). Direct contact with infected wild birds or indirect exposure through polluted settings are examples of potential pathways [10]. Although this option cannot be completely ruled out, it is noteworthy that there is no conclusive genomic or epidemiologic evidence linking wild birds to cattle as the main source of transmission.

Shared equipment and human movement have been recognized as potential routes for intra-herd transmission, which is a worry [8]. Since the virus has been found on milking equipment, it is possible that poor sanitation procedures could make it easier for cows to infect one another [11].

A new H5N1 variation known as D1.1 was discovered in Nevada dairy cattle in February 2025 [12]. This mutation suggests numerous independent spillover occurrences from avian reservoirs to cattle, which is different from the previously common B3.13 lineage [13]. The appearance of D1.1 highlights the virus’s dynamic character and the need for constant monitoring [8]. There have been documented human H5N1 cases connected to exposure to dairy animals [8]. A human infection in Texas linked to suspected contact with diseased dairy cows was confirmed by the Centers for Disease Control and Prevention (CDC) in April 2024 [8]. There have been occasional cases since then, mostly among those who have had close contact with the afflicted cattle [10]. There are serious public health issues when H5N1 is found in dairy animals [14]. The presence of H5N1 in raw milk presents possible dangers even when pasteurization successfully inactivates the virus [10]. To guarantee the safety of the commercial milk supply, the U.S. Food and Drug Administration (FDA) and the U.S. Department of Agriculture (USDA) have conducted outreach to the dairy industry and released guidelines highlighting the significance of pasteurization [15].

In order to reduce the risk of transmission and protect the health of both humans and animals, the introduction of H5N1 into dairy cattle populations is a complicated epidemiological problem that calls for concerted efforts in surveillance, biosecurity, and public health interventions [8].

The H5 subtype influenza virus is the most frequently encountered avian influenza virus, causing numerous illness outbreaks in domestic poultry and wild birds globally. The clade 2.3.4.4 HA of H5 viruses is further divided into eight subclades, labeled 2.3.4.4a to 2.3.4.4h. Over the last three years, H5 viruses with the clade 2.3.4.4b HA gene have garnered significant attention. Early in 2020, H5N8 viruses containing the clade 2.3.4.4b HA gene infected domestic poultry and wild birds, resulting in the death of approximately 33 million domestic birds across Europe, Africa, and Asia. Additionally, these H5N8 viruses reassorted with other avian influenza viruses, leading to the emergence of H5N1, H5N2, H5N3, H5N4, H5N5, and H5N6 viruses. Since their detection in the Netherlands in October 2020, H5N1 viruses with the clade 2.3.4.4b HA gene have spread to numerous countries in Europe, Africa, Asia, and the Americas. Between September 2021 and March 2022, H5N1 viruses containing the clade 2.3.4.4b HA gene were identified in wild birds and domestic poultry in China during routine surveillance [13].

HPAI A(H5N1) clade 2.3.4.4b viruses have spread rapidly among wild birds worldwide from 2020 to 2021, causing outbreaks in poultry and other animals. Epidemiological data from EMPRES-i indicate that between January 2020 and March 2022, +7778 outbreaks were caused by various H5 HPAI viruses containing the clade 2.3.4.4 HA gene. Specifically, H5N1 viruses caused 4284 outbreaks, H5N2 caused 62, H5N3 caused 15, H5N4 caused 14, H5N5 caused 158, H5N6 caused 24, and H5N8 caused 3221 outbreaks, demonstrating a shift in the major strains causing global avian influenza outbreaks from H5N8 to H5N1 since October 2021 [13].

From 2 December 2023 to 15 March 2024, outbreaks of HPAI A(H5) were documented in 227 domestic and 414 wild birds across 26 European countries. Most HPAI outbreaks in poultry were attributed to virus transmission from wild birds. HPAI viruses have led to substantial mortality in commercial poultry, causing significant economic losses worldwide. The HPAI H5 or H7 virus generally infects chickens, with fatality rates ranging from 90% to 100% [8]. On 30 October 2024, the USDA confirmed that a pig in Crook County, Oregon, tested positive for the H5N1 avian flu virus—marking the first known case of H5N1 in pigs within the United States. Of the other pigs on the farm, two are currently awaiting test results, while two others have tested negative. Notably, the pig that tested positive displayed no symptoms of illness. This finding raises concerns since there is a chance of genetic reassortment when pigs contract influenza viruses from both humans and birds. The risk to the public’s health could be exacerbated if this mixing of virus strains in pigs results in new versions that are more capable of infecting humans. Therefore, continuous testing, biosecurity protocols, and surveillance are essential for situation monitoring and management [5].

The first natural A(H5N1) infection in ruminants was reported in goat kids in the United States [14]. A(H5N1) viruses have also been detected in barn cats, birds, and other animals such as raccoons and opossums that resided in or around human settlements or died in affected areas [15]. In June 2023, cats in Poland tested positive for the HPAI H5N1 virus, with contaminated cat food being a suspected source of infection. Similar infections in cats were also reported in Germany, the United States, and South Korea [16]. In Canada, a dog was confirmed to contract the virus in 2023 after chewing on a wild goose. These infections in companion animals may pose health risks to their owners and veterinarians. In 2004, two tigers and two leopards in a Thai zoo were infected with the H5N1 HPAI virus after consuming raw chicken likely contaminated with the virus [17].

In 2022, minks on a farm in Spain tested positive for the HPAI H5N1 virus, with lung lesions observed upon further examination. In Finland, an otter was found dead due to HAPI H5N1 virus infection, with microscopic examination revealing meningoencephalitis in the brain. Dolphins and sea lions in Peru were found positive for HPAI H5N1 in 2022, with affected animals either dead or exhibiting respiratory and/or neurological symptoms [18].

The USDA recently reported a multistate outbreak of A(H5N1) viruses in dairy cows, raising significant national and global concern. It was recently announced that commercially sold milk in 10 states contained fragments of the H5N1 influenza virus [19]. The American Association of Bovine Practitioners (AABP) now recommends naming the virus Bovine Influenza A Virus (BIAV) [20].

In addition to being a major animal health emergency that has resulted in huge production and financial losses, the ongoing spread of H5N1 among dairy cattle also poses a serious risk to public health because of the possibility of occupational exposure on dairy farms. The uncommon interspecies transmission of H5N1 clade 2.3.4.4b to several mammalian species, including cattle with the B3.13 genotype, broadens our knowledge of influenza A virus adaptation in birds. Concern for other mammalian hosts, including pigs, domestic cattle, pets, and people, is increased by this unanticipated route of transmission.

Even though there have only been a few and mild human cases in the United States thus far, there is still a risk because H5N1 is still spreading throughout the region and reassorting with other avian and mammalian influenza subtypes, which might make it more transmissible or pathogenic [5].

Further compounding the concern, researchers at Kyushu University recently identified blowflies—a species attracted to decaying organic material and feces—as potential carriers of the avian flu virus in southern Japan. Published in *Scientific Reports*, their findings introduce an important vector of transmission, suggesting that blowflies could spread the virus to poultry and other animals, thereby increasing the risk of farm contamination. This discovery underscores the need for innovative control measures to mitigate avian influenza outbreaks and protect both animal and human health, particularly in poultry and dairy production settings [3].

## 3. Clinical Presentations in Dairy Cows

In dairy cattle, highly pathogenic avian influenza (HPAI) H5N1 often presents as a mild and frequently vague clinical presentation [8]. Common symptoms of affected cows include tiredness and decreased appetite [21]. A decrease in milk supply, occasionally accompanied by thickened or discolored milk, is a noticeable symptom in breastfeeding cows [22]. Additionally, certain calves may exhibit moderate respiratory symptoms such as clear nasal discharge and, on occasion, diarrhea [20]. In some instances, fever and dehydration have been noted [21]. Although herd production may be impacted by these symptoms, it is noteworthy that the overall morbidity in an affected herd is usually minimal, with less than 10% of cows exhibiting symptoms of sickness [23]. Furthermore, the death rate is still low; culling rates of 2% or lower have been observed [22]. Effective detection and management of H5N1 in dairy cattle depend on close observation and timely diagnostic tests because these clinical indications are mild [6].

The first reported instance of influenza in cattle occurred in 1949, when 160,000 cattle were affected in western and central Japan [23]. This outbreak of bovine influenza was short-lived with recovery occurring within 2–3 days. The symptoms described include high fever (40–42 °C), blepharitis, nasal discharge, anorexia, tympanites, pneumonia, joint issues, and a decrease in milk production.

In 1997, dairy cows in Bristol, southwest England, developed an idiopathic disease characterized by intermittent drops in milk production [24]. By 1999, Gunning et al. observed an increase in spontaneous cases of influenza in milking cows, with an annual incidence rate of 10–20% in some herds in England. Symptoms included a sudden decline in milk output, mild fever, anorexia, and occasional respiratory signs such as nasal discharge and increased respiratory rate [24].

The dairy industry now faces significant challenges as the H5N1 virus infects approximately 10% of cows in each afflicted herd. Symptoms include reduced milk output and thicker, colostrum-like milk, which are more severe in older or nursing cows. According to the Texas Animal Health Commission, infected herds account for up to 40% of milk production losses [13].

In late January 2024, production veterinarians reported unexplained decreases in milk output, decreased feed intake, and changes in milk quality in dairy cattle [25]. Gunning et al. utilized HI tests to determine the prevalence of influenza A virus (IAV) infections in cattle. Of the 40 cattle tested, 60% had elevated antibody titers to the H1N1 virus, while 65% had elevated antibody titers to the H3N2 virus. Crawshaw et al. further demonstrated the correlation between elevated titers to H1N1 and H3N2 viruses and lower milk output in cattle. The loss of milk output (the difference in mean milk production between uninfected and infected animals) was calculated to be 159.9 L, accounting for a 2% reduction in lactation yield per cow.

In this context, the impact of the H5N1 virus on cattle, resulting in a significant decline in milk supply, is not entirely unprecedented but represents a serious concern for the dairy industry.

## 4. Human Infections and Occupational Risks

Sporadic human infections with the HPAI A(H5N1) virus have been reported in 23 countries over the last two decades, with a wide range of clinical severity and a cumulative case mortality rate of more than 50%. According to monitoring systems by the CDC and other government organizations, animal interaction is the only demonstrated mode of HPAI transmission to humans [25]. There have been reports of HPAI A(H5N1) virus detections in mammals in over 20 states in the US [8].

Workers who may be exposed include, for example, the following: farmers and other livestock workers who raise poultry, dairy products, and other types of livestock; veterinarians and their personnel; responders in the fields of animal health and public health; dairy laboratory employees; food processing employees who handle raw milk and other verified or possibly contaminated items; and slaughterhouse employees who carry out specific duties on nursing dairy animals [8].

The WHO provides accurate information on avian influenza, stating that direct contact with diseased animals (through handling, culling, slaughtering, or processing) or indirect contact (via environments contaminated with sick animals’ bodily fluids) poses a risk of human infection.

The genetic sequencing of the A(H5N1) virus from affected cattle and the farm worker identified clade 2.3.4.4b, which has been reported in wild birds, commercial poultry, backyard flocks, and other animals in the United States since January 2022 [8]. On 1 April 2024, the Texas Department of State Health Services, following CDC confirmation, reported that a commercial dairy farm worker tested positive for HPAI A(H5N1) virus infection via real-time reverse transcription–polymerase chain reaction (RT-PCR) after exposure to dairy cattle suspected of being infected. The CDC validated these laboratory findings using RT-PCR and sequencing.

On 22 May 2024, the Michigan Department of Health and Human Services reported one A(H5) case in a dairy farm worker on a farm confirmed to have A(H5N1) virus in cattle. This individual, enrolled in an active text-based monitoring program, reported only eye symptoms. Avian influenza viruses can infect and cause disease in humans, ranging from mild flu-like symptoms or eye inflammation to severe, acute respiratory disease and/or death. The case fatality rate for A(H5) and A(H7N9) subtype virus infections in humans is higher than that of seasonal influenza infections. 

With the growth of global travel, a pandemic can spread rapidly, leading to significant health, economic, and social consequences [26,27]. A systematic review of the literature on personal protective equipment (PPE) knowledge, attitudes, and practices revealed that animal producers often fail to adopt preventive measures and may not always perceive zoonoses as a threat [27]. Assessment of existing infectious disease risk assessment tools revealed that none fully and directly integrate human infectious disease prevention. The findings highlighted strengths and shortcomings in zoonotic disease prevention knowledge, attitudes, and behaviors, and identified components that can be addressed to foster a shared understanding among dairy farm supervisors and workers [28].

The key measure for occupationally exposed workers to AI is the implementation of biosecurity practices, with an emphasis on the provision and effective use of PPE. Educational measures are also crucial and should be implemented through training and educational programs [29].

## 5. The Impact of H5N1 on Milk Safety and Public Health

Given that many affected dairy producers have reported deaths among outdoor domestic cats that consumed raw milk from infected cows on the farm, which coincided with the illnesses in cattle, the risk of HPAI H5N1 foodborne transmission via milk and dairy products from affected herds during this outbreak is especially concerning [7]. It has been shown in a mouse study that milk from infected cows may help spread H5N1 orally. In this study, mice were given 50 microliters of milk that contained 3 × 10^6^ plaque-forming units (PFUs) and was spiked with H5N1 [30]. Furthermore, Eisfeld et al. found that mice that received dosages of 25, 10, and 5 µL of raw milk contaminated with H5N1 at a concentration of 1.3 × 10^4^ PFU/mL had survival rates of 40%, 80%, and 100%, respectively [21]. Initial investigations of raw milk samples from sick animals indicated that the virus had a strong preference for mammary tissue, with significant viral shedding into raw milk, sometimes reaching levels of 10^8^ log^10^ 50% tissue culture infectious dose (TCID50). Several factors ultimately influence the potential public health risk associated with consuming dairy products made from milk containing H5N1. These factors include the initial viral load, the persistence of H5N1 in raw milk, the effectiveness of processing methods like pasteurization in inactivating the virus, and human susceptibility to infection and the infectious dose [31]. Additionally, dairy farm workers who come into contact with contaminated unpasteurized milk during milking are at considerable risk, which could lead to an increase in H5 infections and allow the H5 virus to adapt in humans, potentially enabling person-to-person transmission. Importantly, milking frequently occurs at human eye level, with the human workstation being physically lower than the cows, increasing the risk of infected milk contact with mucous membranes [32]. Early studies of bovine-associated H5N1-contaminated milk reveal that while viral loads drop over time, infectious viruses can remain for more than 5 weeks when refrigerated at 4 °C [33]. This study shows that the H5N1 strain from the outbreak can remain stable at low temperatures for extended periods of time. Other research and simulations based on nonbovine H5N1 and influenza strains have also discovered that lower temperatures limit the rate of viral breakdown [34]. Early studies of bovine-associated H5N1 on materials associated with dairy milking equipment discovered that the virus remained active for several hours, emphasizing the importance of cleaning and sanitization of equipment between raw milk loads at processing facilities to prevent cross-contamination and protect worker health [35]. PCR surveys of commercial pasteurized milk and bulk raw milk tanks have detected fragments of H5N1, suggesting that such events are common [33]. Milk must be pasteurized at 63 °C for 30 min for vat pasteurization and 72 °C for 15 s for continuous flow pasteurization. If the milk product contains added sweeteners, has a fat content of ≥10%, or has a TS content of ≥18% (e.g., ice cream mix, heavy cream), the temperature should be increased by 3 °C (USPHS/FDA, 2019). Personal protective equipment such as face shields, masks, and eye protection are required to prevent H5N1 from spreading from dairy cows to humans. To combat the present outbreak, the use of disposable liners or sanitization of liners between cows should help reduce the spread of the influenza virus among animals at a facility [36]. It is important to note that the first risk mitigation step is to exclude diseased cows from contributing to the milk supply slated for commercial processing under the pasteurized milk ordinance requirements [37].

## 6. Diagnostic Methods and Surveillance

The discovery highlights the urgent need to contain the spread of the virus. Early detection is essential for effectively controlling outbreaks in both animals and humans.

Key diagnostic methods include biosensors, molecular, genetic, immunological, and serological detection. Advancements focus on improving convenience, speed, accuracy, and sensitivity. Real-time PCR (RT-PCR) has high specificity and sensitivity, but they require specialized lab equipment and skilled personnel [19].

The CDC recommends monitoring exposed individuals for at least 10 days and conducting RT-PCR testing with H5-specific primers and probes if symptoms appear, in collaboration with health authorities under appropriate biosafety conditions.

### 6.1. Genomic Sequencing and Surveillance

Genomic sequencing of H5N1 RNA from infected specimens is essential to monitor genetic reassortment and identify mutations that could affect transmissibility or resistance to antiviral treatments. Sequencing data from infected cattle, for instance, can reveal transmission patterns and highlight public health risks associated with viral spread. As an example, genomic sequencing during the 2024 epidemic of HPAI H5N1 in U.S. dairy cattle showed that the virus discovered in a pediatric case in California closely matched strains seen in poultry, dairy cows, and wild birds [8]. This discovery raised concerns about possible public health consequences of such cross-species diseases and implied interspecies transmission [8].

Likewise, the discovery of a novel H5N1 variation, D1.1, in Nevada dairy cattle highlighted the value of sequencing in tracking the evolution and dissemination of viruses. This variation was different from the previously identified B3.13 strain, according to genomic study, which suggests that cattle were introduced to the species many times from wild birds [35]. To comprehend transmission dynamics and put into practice efficient management techniques, this information is essential [35].

Sequencing technology has proven essential in tracking the spread of other viruses in animals, in addition to influenza [36]. An analysis of whole-genome sequencing showed that technology could provide enough genetic variation to determine herd-to-herd transmission pathways, which would help guide biosecurity and disease control plans [36].

By detecting possible zoonotic dangers and directing preventive steps, these examples show how sequencing data not only clarify transmission patterns but also influence public health responses.

Furthermore, functional studies that address the biological significance of detected modifications in the viral genome are extremely significant [37].

Early diagnosis of H5N1 in humans is critical for preventing transmission and minimizing severe illness or fatalities. Given the changing risk of H5N1, precautions are essential since some strains can be extremely dangerous to both humans and animals, with the ability to cause serious illness and even death. Monitoring infected patients, carrying out syndromic and laboratory surveillance, and organizing epidemiologic studies are all examples of surveillance activities that are directed by a One Health approach—coordinating human, animal, and environmental health responses. These steps are essential for assessing current defenses, including vaccinations, antiviral medications, and diagnostic testing [19].

### 6.2. Comprehensive Surveillance Networks

More than 100 public health laboratories in all 50 U.S. states use CDC molecular assays for detecting seasonal and novel influenza A viruses, and nine of these sites perform genomic sequencing to characterize the virus. The CDC’s National Syndromic Surveillance Program (NSSP) also gathers emergency department and healthcare data to identify unusual influenza patterns, especially in regions where A(H5N1) has been detected in animals. 

Additionally, the National Wastewater Surveillance System supports these efforts by tracking influenza trends through wastewater analysis, which offers insights into viral circulation in communities.

Interpreting surveillance data for A(H5N1) can be challenging due to overlapping symptoms with other respiratory diseases. To reduce the hazards posed by H5N1 and efficiently respond to possible outbreaks, ongoing efforts are necessary to enhance diagnostic precision, early detection, and worldwide surveillance coordination [8].

## 7. Socio-Economic Analysis and Value Chain Impact of H5N1

The re-emergence of highly pathogenic avian influenza (HPAI) in the United States (US) between 2014 and 2015 led to the largest avian influenza outbreak in US history, with 232 cases and an estimated economic impact of USD 950 million [32]. The epidemic had a significant economic impact on the turkey farming industry: contractual turkey growers experienced an average production loss of 0.9 cycles (38%) alongside a 9.3% reduction in management fees in the eight months following the outbreaks. Consequently, their production and enterprise income decreased by 36% and 39%, respectively. During idle production periods, about 93% of producers did not seek additional work, and 59% had to use an average of USD 3200 from savings to cover labor costs. Furthermore, 62% of producers had considered expanding their operations but postponed these plans due to the outbreak [32].

The H5N1 outbreak also impacted the layer (egg) industry, with losses exceeding USD 60 million from January to August 2006. In Nigeria, where the GDP stood at USD 72.1 billion in 2004, HPAI H5N1 not only imposed financial losses but also affected trade, tourism, and food security. Public misconceptions about the virus led to a reduced availability of animal protein, price surges for alternative products, and increased production costs for livestock farmers [32]. In Egypt, HPAI led to the death or culling of around 36 million chickens, with 1.6 million birds killed between February 2007 and June 2008, plus 277,000 dying across 287 outbreaks, and an additional 2.2 million eggs destroyed on affected farms [32].

In socio-economic evaluations, the extensive impacts of animal illnesses such as H5N1 on the cattle value chain are frequently overlooked. Understanding the value chain’s vertical linkages—including the synergistic, complementary, and competing processes that influence the impact of disease on both the economic and epidemiological levels—is necessary for effective analysis. Value chain analysis can reveal how animal diseases affect complex markets, especially in developing nations, by mapping relationships among stakeholders across the marketing channel. This approach is particularly relevant for livestock systems with extensive distribution channels but remains underutilized in disease impact evaluations, despite its potential to identify vulnerabilities and risk pathways in the marketing system [38].

Business operators in these systems make decisions that affect how various stakeholders cooperate and communicate, which in turn affects how value chains are managed. The value chains are ultimately driven by consumer demand for particular food quality and quantity, highlighting the necessity of these systems to be resilient in the face of disease outbreaks in order to achieve goals for food security and economic stability.

## 8. Management and Prevention

There are presently no approved immunizations for HPAIV H5N1 in dairy cattle in the United States, despite the fact that animal immunization has the potential to indirectly protect humans against newly emerging zoonotic viral infections with a high potential for spread. However, other countries and regions, such as Mexico, China, Egypt, and the European Union, have taken steps to vaccinate against HPAI in poultry and other animals.

A comprehensive One Health approach that acknowledges the interdependence of environmental, animal, and human health is necessary for managing and preventing H5N1 avian influenza. To successfully contain and stop the virus’s transmission, this approach entails concerted efforts across several sectors.

Vigilant surveillance and a timely reaction to possible H5N1 cases are essential for safeguarding human health [8]. Preventive measures are advised by the Centers for Disease Control and Prevention (CDC), which include testing and monitoring of exposed individuals, the use of personal protective equipment (PPE) for those exposed to infected animals, and antiviral medicines as required [8]. Campaigns to raise public knowledge of transmission dangers and preventive measures are crucial. Healthcare professionals should also receive training on how to identify signs and handle cases efficiently [39].

Animal populations must be closely monitored and managed in order to stop H5N1 at its source (FAO, 2024). For early virus detection, surveillance measures must be put in place in cattle and poultry farms [40]. To safeguard the health of both animals and the general public, Michigan’s dairy surveillance program, for example, analyzes milk samples every month to check for the presence of H5N1. To stop the infection from spreading, infected animals should be put in quarantine or put down. Campaigns to boost immunity in animal populations at risk might also be taken into consideration [41]. 

Environmental factors are important in the dynamics of H5N1 transmission. Early diagnosis of newly developing infectious illnesses depends on wildlife health monitoring [42]. Potential epidemics can be detected early with surveillance systems that monitor health trends in wild bird populations [43]. By putting biosecurity measures in place, such as limiting access to farms and disposing of animal waste properly, environmental contamination can be decreased [44]. Avoiding wild bird contact and reporting sick or dead animals are two ways that public education improves environmental health and lowers the risk of transmission [8].

In conclusion, a One Health strategy for controlling and averting H5N1 avian influenza entails coordinated actions from the environmental, animal, and human health domains [45]. Effective control and prevention of the virus’s transmission require cooperative tactics, such as biosecurity measures, public education, and surveillance [8].

The CDC suggests two classes of FDA-approved anti-influenza medications: cap-dependent endonuclease inhibitors (CENIs), including baloxavir, marboxil, and NA inhibitors, including oseltamivir, zanamivir, and peramivir [43].

The acquisition of specific substitutions in the viral NA (e.g., I117T/V, I223T, S247R, S247N, H274Y, and N294S) or PA (e.g., E23G/K/R, A34R, A37T, I38T, and E199G) proteins may result in the emergence of viruses with impaired or reduced susceptibility to available antiviral drugs, even though these drugs remain effective against AIV strains, including the recent HPAIV H5N1 virus [44]. The prevalence of antiviral resistance markers against already licensed NAI and the CENI was recently examined in the sequences of the NA and PA regions of 202 cow HPAIV H5N1 strains from two separate farms in the states of Texas and Ohio [37]. It has been demonstrated that the substitution of NA-T438I (N1 numbering) in one strain lessens the suppression of HPAIV H5N1 by NAI zanamivir and peramivir, but not oseltamivir [43]. Strict biosecurity and management procedures are necessary to stop the spread of H5N1 and related zoonotic viruses. When prepared in accordance with Food and Drug Administration (FDA) rules, pasteurization successfully inactivates viruses, guaranteeing that retail milk is safe for human consumption. Dairy products and unpasteurized milk, however, pose health hazards to both people and animals. Due to the possibility of infection, milk from herds with H5N1 or from cows that may be sick should not be transferred to other farms or animals [45].

Approximately 58% of the 1407 recorded human infections are zoonotic, and 73% of emerging human pathogens originate from animal sources [46]. This underscores the importance of strict biosecurity measures, which have proven effective in disease control on farms. For instance, basic biosecurity interventions successfully suppressed outbreaks of equine influenza and *Rhodococcus equi* on horse farms, highlighting how preventive practices can significantly reduce infection risks [47].

## 9. Key Biosecurity Measures on Farms

1. Animal segregation and quarantine: Clearly defined physical borders between the rearing sites for young, sick, and recently arrived animals help prevent the transmission of illness. To make sure they fulfill health and immunization requirements, new or returning animals should be placed in quarantine, examined, and closely watched. [48].
2. Protective gear and controlled access: Establish zones with different degrees of protection to limit access to animal areas. In order to reduce the possibility of cross-contamination, farm workers should wear clothing and footwear appropriate for each region. This method protects healthy animals and helps identify possible sources of illness.
3. Regular testing of water, soil, feed, and fodder is essential to detect potential sources of contamination. Effective manure management plans covering collection, storage, treatment, and disposal help prevent pathogen spread and support biosecurity objectives [47].
4. Carcass disposal: Biosecurity agencies in countries like Australia, New Zealand, the United States, and Canada recommend composting due to its benefits for routine and emergency mortality management. Properly managed composting prevents environmental contamination and minimizes pathogen exposure to other animals.
5. Pest and wildlife control: Securing entry points to animal housing, pens, and barns limits access by pests, stray animals, and wildlife, which could otherwise introduce or spread disease.
6. Regular cleaning and disinfection are critical components of effective biosecurity on farms. Only certified and internationally recognized disinfectants should be used on agricultural premises. This helps eliminate pathogens from equipment, facilities, and surfaces that animals or personnel frequently contact.
7. Regular health checks, screening, and vaccinations of livestock are vital to maintaining herd health and identifying infections early. Sick animals should be promptly isolated and treated, and detailed health records maintained for effective monitoring [47].

### 9.1. Reducing Public Health Risks from Zoonotic Influenza

The fact that HPAIV H5N1 can continue to develop genetic alterations in a wide range of affected mammalian species is crucial because it may make the virus more contagious to and among humans [7]. To effectively control zoonotic influenza, like H5N1, public health measures must include risk-based pandemic preparedness, comprehensive examination of human cases, and robust surveillance in both animal and human populations. Investigating human instances of zoonotic influenza requires cooperation and information exchange between public health and animal health organizations because coordinated responses enhance outbreak identification and response strategies. Additionally, to evaluate the effects of the unique amino acid changes, functional assays are needed. To prevent further evolution and repeated human–H5N1 contacts, it is imperative to eradicate the virus from cattle. To put an immediate stop to the cow plague, it is not enough to require a negative diagnostic test for IAV when moving cows from one farm to another. There is a real chance that new mammals will serve as long-term reservoirs for HPAIV, especially the H5N1 subtype, due to the ongoing development of IAV. The WHO has emphasized the significance of global proactive viral surveillance systems as a result. To gain a better understanding of the factors causing HPAIV H5N1’s rapid spread and predominance in birds, other wild or domestic species, and new mammalian host species, it is also critical to monitor and carry out research utilizing a One Health approach. 

### 9.2. Public Guidelines and Precautions

1. Avoiding Animal Contact in High-Risk Areas: People should stay away from direct animal contact in places including farms, live animal marketplaces, and slaughterhouses where animal influenza cases are known to occur. Additionally, anyone who are at high risk, such as youngsters, elderly people, pregnant or postpartum women (within six weeks), and people with compromised immune systems, should avoid coming into contact with surfaces contaminated with animal excrement.
2. Hand Hygiene and Food Safety: Hand washing with soap and water is important, especially before and after handling animals or their products. Alcohol-based hand sanitizers are advised in situations where soap and water are unavailable. Practices for food safety should also be strictly adhered to. This entails keeping food preparation facilities clean, cooking meat completely, keeping raw and cooked food separate, and storing meat correctly to avoid cross-contamination.
3. Travel Advice for Outbreak areas: Visitors to places where avian influenza outbreaks are occurring should stay away from live animal markets, poultry farms, and any locations where poultry might be processed. Travelers should immediately alert local health authorities if they return with respiratory symptoms suggestive of zoonotic influenza. Rapid testing, triage, and clinical management, including antiviral therapy and supportive care if necessary, are made possible by early detection and reporting.
4. Avoiding Contact with Sick or Dead Animals: People should tell local wildlife or veterinary authorities about any sick or dead animals they come into contact with, especially wild birds, so that they can be safely removed and investigated.
5. Employers should update or create a workplace health and safety plan to protect workers who may be exposed, perform a site-specific hazard assessment to identify potential exposures based on work tasks and setting, and select controls to reduce or eliminate hazards, such as exposure to HPAI A(H5N1) viruses, using the hierarchy of controls.

### 9.3. Clinical Management and Public Health Reporting

Suspected cases of zoonotic influenza should be reported to health authorities immediately. Effective clinical case management includes assessing the patient’s disease severity, determining risk factors, isolating contagious individuals, and providing appropriate treatment. Antivirals and supportive care are essential for managing severe cases and preventing complications.

### 9.4. Impact of Agricultural Intensification and Climate Change

In addition to globalization, resource depletion, and climate change, the intensification of livestock agriculture to satisfy the world’s demand for animal-based protein has increased the risk of the spread of zoonotic diseases. To protect public health, reduce financial losses, and guarantee food safety, controlling zoonotic diseases in the livestock industry is crucial. To effectively prevent, identify, and respond to animal illness outbreaks, significant resources are needed, particularly in poor nations [47].

In conclusion, strong surveillance, interagency cooperation, and adherence to food safety and hygiene protocols are essential for lowering the dangers to public health posed by zoonotic influenza. Together with effective public health response systems, these preventative strategies aid in halting the spread of zoonotic illnesses and safeguarding both human and animal populations.

## 10. Conclusions

Monitoring and controlling the spread of H5N1 in both animal and human populations require constant, attentive study and surveillance. Reducing the impact of this virus requires quick identification and action in response to any new threats to human health. To effectively manage and contain the current H5N1 avian influenza outbreak in U.S. dairy cattle, proactive surveillance, early detection, and coordinated efforts among local, state, and federal health agencies—as well as among livestock producers, dairy distributors, and other stakeholders—are essential.

A One Health approach, which recognizes the interconnectedness of human, animal, and environmental health, is fundamental to effective control and prevention strategies for avian influenza. By fostering collaboration between public health, veterinary, and environmental agencies, the One Health framework enables a more comprehensive and coordinated response, addressing the various transmission pathways and ecological factors involved in H5N1 outbreaks.

Dairy cattle must be tested for the influenza A virus at a National Animal Health Laboratory Network (NAHLN) facility that has been accredited to lower the risk of illness transmission between states. Only animals that test negative are allowed to cross state lines. Herd owners must submit comprehensive epidemiological information, including movement history, to enable tracking and containment efforts when cattle meant for interstate movement test positive. To guarantee adherence to biosecurity regulations and stop additional spread, dairy calves slated for interstate transportation must also fulfill conditions set by the Animal and Plant Health Inspection Service (APHIS) [19].

These combined efforts underscore the importance of integrated approaches to surveillance, biosecurity, and regulatory compliance, fostering a robust response to the H5N1 outbreak and safeguarding both animal and public health through the collaborative One Health approach.
